# New Analysis Framework Incorporating Mixed Mutual Information and Scalable Bayesian Networks for Multimodal High Dimensional Genomic and Epigenomic Cancer Data

**DOI:** 10.3389/fgene.2020.00648

**Published:** 2020-06-18

**Authors:** Xichun Wang, Sergio Branciamore, Grigoriy Gogoshin, Shuyu Ding, Andrei S. Rodin

**Affiliations:** Department of Computational and Quantitative Medicine, Beckman Research Institute and Diabetes and Metabolism Research Institute of the City of Hope, Duarte, CA, United States

**Keywords:** The Cancer Genome Atlas, Bayesian networks, multimodal big data, variable selection, mixed mutual information, methylation, genomic and epigenomic molecular data

## Abstract

We propose a novel two-stage analysis strategy to discover candidate genes associated with the particular cancer outcomes in large multimodal genomic cancers databases, such as The Cancer Genome Atlas (TCGA). During the first stage, we use mixed mutual information to perform variable selection; during the second stage, we use scalable Bayesian network (BN) modeling to identify candidate genes and their interactions. Two crucial features of the proposed approach are (i) the ability to handle mixed data types (continuous and discrete, genomic, epigenomic, etc.) and (ii) a flexible boundary between the variable selection and network modeling stages — the boundary that can be adjusted in accordance with the investigators’ BN software scalability and hardware implementation. These two aspects result in high generalizability of the proposed analytical framework. We apply the above strategy to three different TCGA datasets (LGG, Brain Lower Grade Glioma; HNSC, Head and Neck Squamous Cell Carcinoma; STES, Stomach and Esophageal Carcinoma), linking multimodal molecular information (SNPs, mRNA expression, DNA methylation) to two clinical outcome variables (tumor status and patient survival). We identify 11 candidate genes, of which 6 have already been directly implicated in the cancer literature. One novel LGG prognostic factor suggested by our analysis, methylation of TMPRSS11F type II transmembrane serine protease, presents intriguing direction for the follow-up studies.

## Introduction

The Cancer Genome Atlas (TCGA) resource contains genomic data compiled for more than 30 different types/subtypes of cancer (Tomczak et al., 2015). For each type, clinical outcome/progression data (e.g., tumor status and patient survival) for a considerable number of patients is matched to the large-scale molecular data. The latter is multimodal, ranging from genetic (e.g., somatic mutations) to expression (e.g., RNA-seq gene expression) to epigenetic (e.g., promoter methylation) data. Not surprisingly, there is substantial enthusiasm for causally linking the latter to the former using various modeling and secondary data analysis techniques (Jeong et al., 2015; Phan et al., 2016; Hou et al., 2018; Tian et al., 2018; Xu et al., 2018). The ultimate goals of these analyses are (i) to gain better mechanistic understanding of the underlying molecular biology of cancer, primarily by identifying important genes and their interactions; (ii) to construct compact and efficient clinical predictors (e.g., prognostic scores, indices and signatures); (iii) to associate the latter with the particular patient groups and subgroups, in the context of personalized/precision medicine. One of the more attractive and popular methods for such multivariate analysis is Bayesian networks (BNs) (Heckerman, 1995), a well-established fixture in computational systems biology (Friedman et al., 2000). Among the BN advantages are their probabilistic nature, model flexibility, ability to handle non-additive, higher-order, interactions, and ease of the result interpretation. However, applications of BNs to the TCGA (and TCGA-like) data (Gevaert et al., 2006; Xu et al., 2012, 2014; Wang et al., 2013; Huang et al., 2015; Zhu et al., 2015; Kaiser et al., 2016; Wu et al., 2017) face two principal difficulties: combining mixed data types in a single analysis framework, *and* achieving sufficient (for genomic data) scalability, *simultaneously*. (These, of course, are the two fundamental, and interconnected, BN modeling challenges in general, not just in the TCGA application). The latest developments in addressing these two challenges encompass more efficient computational approaches (Gogoshin et al., 2017; Ramsey et al., 2017), and mathematically rigorous and robust methods for handling mixed data, such as mixed local probability models and/or adaptive discretization (Gogoshin et al., 2017; Andrews et al., 2018; Sedgewick et al., 2018). Nevertheless, resolving both difficulties simultaneously in a generalizable toolkit (seamlessly applicable, for example, across the individual TCGA datasets) remains elusive. A promising approach to devising such a toolkit would be to precede the comparatively exhaustive NP-hard BN modeling with a variable selection procedure [for example (Zhang et al., 2014)], where the full dataset is pared down to a subset of variables most relevant to a particular clinical outcome or phenotype. While alleviating the scalability issue, this, however, could potentially “throw away the wheat with the chaff,” especially if the variable selection process (Blum and Langley, 1997; Guyon and Elisseeff, 2003) is of a simplistic and overly too restrictive kind (e.g., a statistically conservative univariate filter). There are three possible ways to address this, namely: (i) increase the scalability of the BN modeling to genomic data levels (possible, but impractical for frequent/serial analyses), (ii) incorporate higher-order interactions into the variable selection step (thus “upgrading” it from the simple filter to the wrapper [Kohavi and John, 1997; Guyon and Elisseeff, 2003; Leng et al., 2010) — this is the solution implemented in Zhang et al. (2014)], or (iii) adjust the transition boundary between the variable selection step and the BN modeling step, depending on the investigators’ computational resources and the nature (dimensionality, sparseness, heterogeneity) of the actual data. It is the third analytical strategy that we propose in this study, with the goal to achieve the optimal compromise between the computational practicality and modeling exhaustiveness.

In our analysis pipeline, we start with the variable selection procedure based on the mixed-type Mixed Mutual Information (MMI) forward selection filter. We compute the MMI values for all available gene-outcome (specifically, tumor status and patient survival) pairs, and use the MMI frequency distribution to select top variables/genes (or, alternatively, to remove bottom variables/genes) before moving on to the BN modeling. This mixed-type measure-based approach to gene selection is the principal innovation of this paper. We then use the maximum entropy (ME) – based discretization to construct the mixed-type BNs using our previously reported scalable BN modeling algorithm and software (Gogoshin et al., 2017). Subsequently, we concentrate on the sub-networks centered around the clinical outcome variables of interest, and identify the molecular gene components belonging to these sub-networks.

The proposed analysis strategy has been applied by us to 12 different TCGA cancer datasets. This allowed us to check for robustness, scalability and generalizability. Here, we present the results for the Brain Lower Grade Glioma (LGG), Head and Neck Squamous Cell Carcinoma (HNSC) and Stomach and Esophageal Carcinoma (STES) datasets (all three datasets being reasonably well-populated and proportionally balanced across the different outcomes and molecular data types). For the purposes of this particular analysis, we decided to concentrate on three types of molecular data, one discrete (somatic mutations) and two – continuous (RNA-seq gene expression, and promoter methylation). This selection is reflective of the recent trends in multimodal cancer data analyses (Zhang et al., 2014; Yoo et al., 2017), makes sense in the broad cancer genetics context (Phipps et al., 2016; Fang et al., 2017; Liang et al., 2017; Rajesh et al., 2017; Zhang C. et al., 2017; Koch et al., 2018), and underscores the comparative importance of the methylation molecular data (Koch et al., 2018). While focusing solely on the gene-centric modalities is inherently limiting (many disease-linked SNPs are localized in the non-coding regions), one of the primary purposes of this study was to showcase the MMI approach (enjoining three different modalities in a single measure/score), which necessitated the gene-centric analysis. In future, we plan to generalize our analytical framework to other, non-gene-centric, data.

We conclude by identifying a compact list of genes potentially associated with cancer-related clinical phenotypes (tumor status and patient survival), scrutinizing these genes in light of the current literature, and discussing the generalizability of our approach to the different datasets, diseases and molecular data types.

## Materials and Methods

### Data Preprocessing

The Cancer Genome Atlas, LGG, HNSC, and STES datasets were downloaded for the clinical data [“Clinical_Pick_Tier1 (MD5)”], SNP data [“Mutation_Packager_Calls (MD5)”], expression data [“mRNAseq_Preprocess (MD5)”] and promoter-centric methylation data [“Methylation_Preprocess (MD5)”]. Patients were further subdivided into (i) two disease progression categories (according to the “tumor status” variable), and (ii) two patient survival categories (high death risk, with survival less than 2 years, and low death risk, with survival more than 2 years, which is a common cutoff point in recent cancer literature). We further excluded patients with ambiguous or missing outcome variable values (e.g., no survival status, survival status as “living” with survival time less than 2 years, tumor status neither “tumor-free” nor “with tumor,” etc.). These clinical variables (“tumor status” and “2-year survival”) were subsequently used for the variable selection purposes, and, eventually, to extract “tumor status” and “survival” – centered sub-networks from the full BNs. Expression data and methylation data (designated by “E” and “M” below, for brevity) were not discretized at this stage, as both variable selection and BN construction tools in our computational pipeline can, by design, accept mixed (continuous and discreet) variable types. SNP (somatic mutation) data (designated by “S” below) were compressed into a binary variable (presence or absence of at least one non-synonymous mutation in at least one sample of the particular gene).

After filtering out patient records with incomplete, partially missing, or ambiguously labeled data, the final datasets consisted of 4782 genes (LGG), 12516 genes (HNSC) and 16164 genes (STES). 273 patient records were available for LGG/tumor status analysis (140 patients with tumor, 133 without); 213 patients – for LGG/survival (120 patients with survival less than 2 years, 93 with long-term survival). Similarly, 260 patient records were available for HNSC/tumor status analysis (94 patients with tumor, 166 without); 139 patients – for HNSC/survival (40 patients with survival less than 2 years, 99 with long-term survival). Finally, 403 patient records were available for STES/tumor status analysis (147 patients with tumor, 256 without); 258 patients – for STES/survival (191 patients with survival less than 2 years, 67 with long-term survival).

Here we would like to re-emphasize that it is possible to include other different molecular data types and outcome variables, both continuous and discrete, into the proposed framework without substantial alterations to the analysis pipeline, except for some rudimentary data preprocessing.

### Variable Selection

There are very few BN algorithms/software solutions that scale up to (epi)genomic levels (tens to hundreds of thousands of variables) (Gogoshin et al., 2017; Ramsey et al., 2017). Even with these, exhaustive analyses require dedicated hardware and weeks of processing time. This might be acceptable for a one-off, “final” analysis, but is clearly impractical for the exploratory research. This is why it is a common practice to carry out variable selection (or feature selection, or feature set reduction) in order to generate a comparatively compact subset of variables to be subsequently fed into the network modeling algorithm/software (Guyon and Elisseeff, 2003). Variable selection approaches range from the very simple (univariate filters) to increasingly more sophisticated; at some point, the latter become essentially indistinguishable from the multivariate modeling methods *per se*. Depending on the dataset to be analyzed, different “couplings” of variable selection and multivariate modeling methods might prove to be more or less effective, and it is difficult to devise *a priori* the objectively optimal combination for each new dataset. For a principally network-centric data analysis approach (innate to the systems biology), it would make sense to feed as many variables into the network-building module as possible, thus “delegating” the resolution of the higher-order/non-additive interactions and conditional independence relationships to the BN algorithm itself. Therefore, for the exploratory research, we suggest that the investigators first define the upper BN scalability limit that they are comfortable with (given the available software/hardware), and then adjust the variable selection cutoff point accordingly. For more “finalized” analysis, that limit should be raised higher (and the variable selection process, consequently, be made less restrictive).

In TCGA dataset (and other similar (epi)genomic resources), there are tens of thousands of potentially predictive/relevant variables (roughly proportional to the number of genes in the human genome). The “hand off” point between the variable selection and BN analysis steps should therefore vary between 100s of variables (for the exploratory and preliminary analyses) and 1,000s of variables (for the final analyses). The actual number might also depend on the shape of the variable selection curve, or on the statistical significance criteria–we stop adding increasingly less significant variables during the forward variable selection process (or stop removing increasingly more significant variables during the backward variable elimination process) when a certain statistical significance cutoff point is reached (Rodin et al., 2009). The above considerations were taken into account in the course of this study, as detailed in the section “Results” below.

It is difficult to integrate the multimodal, mixed-type, data into the variable selection process (filter or wrapper) as, until recently, there has been a paucity of the usable mixed-type metrics. In this study, a recently developed measure, Mixed Mutual Information (MMI) (Gao et al., 2018), was used to link the gene information (a mixed-type vector consisting of the S, E, and M molecular data components for each gene) to the clinical variable (tumor status or 2-year survival) in a “forward-selection-filter” variable selection procedure. MMI is a non-parametric and distribution-free measure [which makes it more attractive than the alternatives, such as linear correlation – especially in the biological networks context (Margolin et al., 2006; Asur et al., 2007)] that is based on the entropy estimates from k-nearest neighbor (k-NN) distances (Kraskov et al., 2004). It is, therefore, sensitive to the choice of the *k* parameter. Lower values of *k* (1–4) tend to lead to higher dispersion, while much higher values (>20) are associated with unnecessarily increased computational complexity and possible overfitting [, personal communication from Gao et al. (2018)]. We have evaluated different values of *k* on the actual TCGA datasets by measuring the Jaccard index for the pairs of consecutive (in *k*) post-selection variable sets as a function of *k*. The index appeared to stabilize in the 8–20 range in 12 different TCGA datasets analyzed (see section “Results” below); therefore, *k* was set at 15 throughout this study.

### Bayesian Networks Modeling

Bayesian networks modeling, in its basic form, reconstructs a sparse graphical representation of a joint multivariate probability distribution of random variables from a “flat” dataset. Nodes in the network represent random variables, edges – dependencies. Absence of an edge between the two nodes indicates conditional independence between them. Recent work in BN methodology refinement led to significant progress in scalability – our latest BN modeling software implementation (Gogoshin et al., 2017) easily processes datasets up to ∼ 1 mln variables × 1 mln datapoints. Handling mixed variable types (both continuous and discrete, in a typical application) is still not entirely seamless; it was recently suggested (Gogoshin et al., 2017; Andrews et al., 2018) that adaptive discretization (of continuous variables) might be preferable to forcing mixed local probability models. Consequently, we were using maximum entropy – based three-bin discretization throughout this study – expression data (“E” molecular data component) and methylation data (“M” molecular data component) were discretized into three bins – which has attractive mathematical properties, and has been shown by us earlier to maintain near-optimal over/under-fitting balance (Gogoshin et al., 2017).

Detailed description of the BN methodology in general and of our implementation (including applications to other types of high-dimensional biological data) in particular can be found in Gogoshin et al. (2017), Zhang X. et al. (2017); here we will only note that (1) our BN implementation uses a hybrid “sparse candidates” + “search-and-score” graduate descent algorithm coupled with various model scoring metrics and maximum entropy-based adaptive discretization; (2) in the resulting BN visualizations, numbers next to the edges and edge “thickness” indicate relative edge strengths (the numbers are the model scores’ ratios for the models with/without corresponding edges, which are proportional to the marginal likelihood ratios); (3) directionality in the network (arrow points attached to the edges, when present) does not necessarily imply the causality flow, and is used predominantly for the mathematical convenience (to avoid cyclic dependencies); (4) when deciphering conditional dependence and independence patterns, it is useful to concentrate on the immediate Markov neighborhood (MN) of a particular variable of interest (such as a clinical outcome). This neighborhood can be roughly defined as all the nodes that are in immediate contact with (“one degree of separation” from) the node representing the aforementioned variable of interest. Under certain conditions, given its MN, the variable of interest is conditionally independent of the remaining variables (rest of the network). Therefore, deriving a MN for a variable of interest is analogous to the variable selection activity, specifically of the embedded variety (Guyon and Elisseeff, 2003). The central step in our computational analysis pipeline is using full BN reconstruction to generate the MN for the clinical outcome variable, and then ascertaining the interplay of the (small number of) gene-related variables (S,E and M molecular data components) within that MN. (It should be noted that MN is a simplification of the more rigorous concept of Markov Blanket – meaning, for our purposes, that sometimes “two degrees of separation” are needed for encapsulating a variable/node of interest).

## Results

**FIGURE 1:**
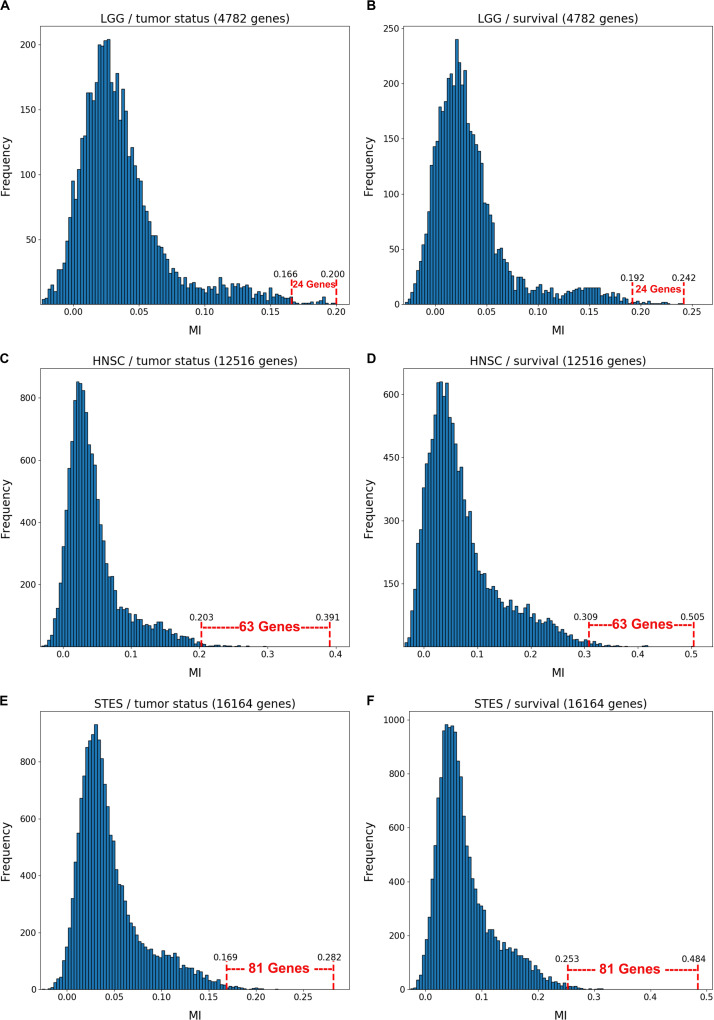
Variable selection process for six combinations of two clinical outcome variables (“tumor status” and “survival”) and three TCGA cancer datasets (LGG, HNSC, STES). MMI between the (S,E,M) molecular data vector and tumor status/survival was computed for 4782 genes (LGG), 12516 genes (HNSC) and 16164 genes (STES). The histogram representation of the MMI distribution is shown with the selection of “top” (i.e., with the MMI CDF >99.5%) genes superimposed on the right tail of the MMI frequency distribution. **(A)** LGG/tumor status; **(B)** LGG/survival; **(C)** HNSC/tumor status; **(D)** HNSC/survival; **(E)** STES/tumor status; **(F)** STES/survival.

Figure 1 depicts the variable selection process for six possible combinations of two clinical variables (“tumor status” and “survival”) and three TCGA cancer datasets (LGG, HNSC, and STES). MMI (mixed mutual information) between (S,E,M) and tumor status/survival was computed for 4782 genes (LGG), 12516 genes (HNSC), and 16164 genes (STES). (All six gene lists, with corresponding MMI values, are available in Supplementary Tables S1–S6). The histogram representation of the MMI distribution, as shown in Figure 1, is convenient, as it allows to evaluate (both visually and quantitatively) the relative predictive values of the top-ranking genes with respect to the outcome variable classification. For the purposes of this study, and to make the resulting full BNs “observable,” we have chosen the “top genes” cutoff value of 99.5% MMI CDF (cumulative distribution function), which leads to the selection of 24 genes (72 future BN nodes/variables in total, comprising 24 S, 24 E, and 24 M components) out of 4728 for two LGG networks, 63 genes (189 nodes/variables) out of 12516 for two HNSC networks, and 81 genes (243 nodes/variables) out of 16164 for two STES networks. Note that the S, E, and M components of each gene vector were considered as the separate nodes/variables in the subsequent BN construction, as at this time we do not have a BN scoring function that can incorporate mixed multivariate distance measures. It should also be noted that although MMI, intuitively, should not be negative, due to the way it is computed it can get into the negative range when (i) continuous variables are involved, and (ii) the number of dimensions is more than two (four, in our case). This said, all the negative MMI values in Figure 1 reside well within the allowed algorithmic negative deviation range, and should not influence the variable rankings [personal communication from Gao et al. (2018)].

Interestingly, every histogram in Figure 1 has a heavy right tail, which sometimes appears to follow a clear “knee point” – for example, at MMI ∼ = 0.08 in Figures 1A–C. This suggests that MMI >0.08 could also be used as a “natural” cutoff value, at least in these three datasets.

**FIGURE 2:**
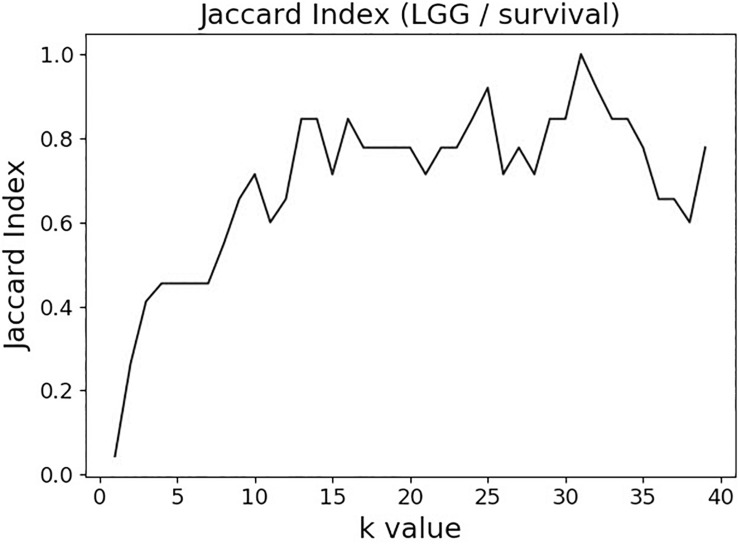
Jaccard index (JI) (“Intersection over Union”) comparing the gene/variable sets resulting from the LGG/survival dataset variable selection with the cutoff set at 99.5% MMI CDF. JI(*k*) compares the sets obtained with *k* and *k+1*.

The variable selection distributions shown in Figure 1 were derived with the MMI parameter *k* set at 15. Figure 2 illustrates the motivation behind that choice, using the LGG/survival dataset example. Shown is the plot of the Jaccard index (JI, a.k.a. set “Intersection over Union,” which is a common measure of sample set similarity) comparing the gene/variable sets resulting from the above variable selection procedure, with cutoff set at 99.5% MMI CDF, where JI(*k*) compares the sets obtained with *k* and *k+1*. It is clear that as k reaches ∼15, the set composition somewhat stabilizes; further increase in k does not seem to offer any advantages. (JI plots for the other datasets exhibit a similar pattern).

**FIGURE 3:**
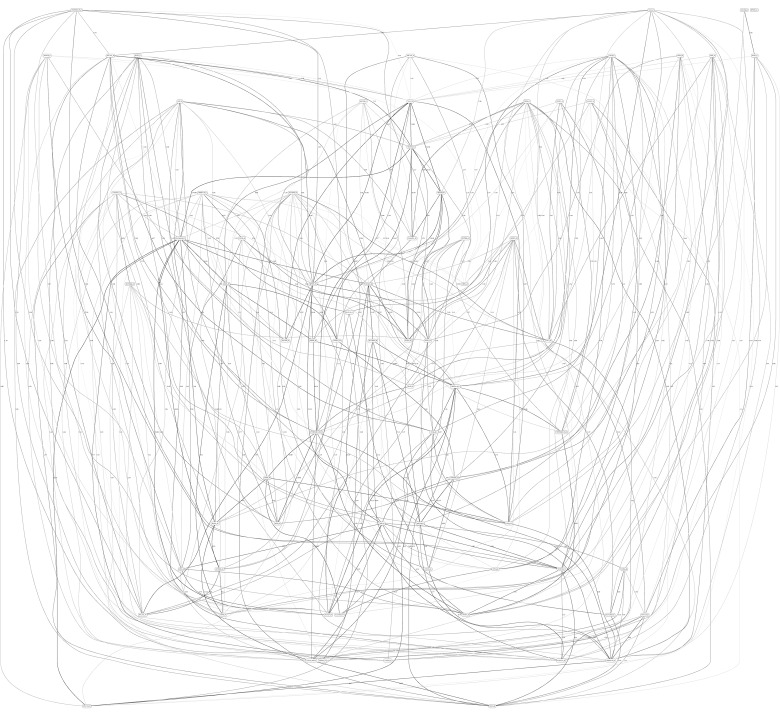
Full BN derived from the LGG/tumor status data. “Tumor_Status” node in the BN is self-explanatory. Other nodes in the networks correspond to the genes/molecular components (gene name_S/E/M). Edges in the network correspond to the dependencies between the nodes. Directionality of the edge (arrow) is for mathematical convenience only and does not imply causation. “Boldness” of the edge is proportional to the dependency strength, also indicated by the number shown next to the edge. Note that the BN pdf image files are searchable (using gene manes), and that all the BN pdf and source files (in DOT format) are included as part of the Supplementary Material.

**FIGURE 4:**
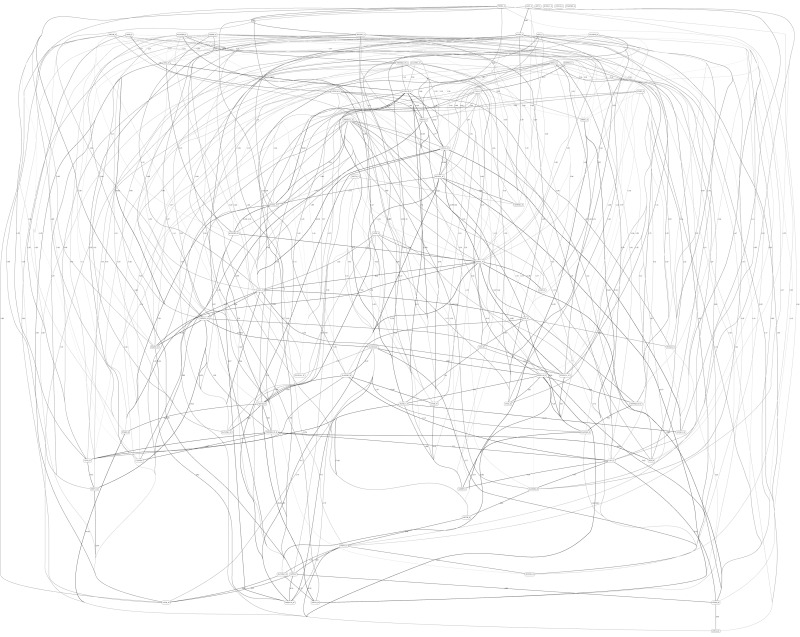
Full BN derived from the LGG/survival data. “Survival” node in the BN is self-explanatory. Other designations are as in Figure 3.

Figures 3, 4 depict the full BNs obtained from the LGG/tumor status and LGG/survival datasets. Supplementary Data Sheets S1–S6 depict, in PDF format, the full BNs obtained from the LGG/tumor status, LGG/survival, HNSC/tumor status, HNSC/survival, STES/tumor status, and STES/survival datasets, respectively. Six corresponding DOT (standard network/causal graphical models format) files can be found in the Supplementary Tables S7–S12.

**FIGURE 5:**
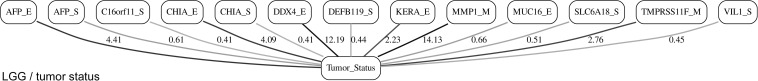
MN of the “Tumor_Status” node in the LGG/tumor status BN.

**FIGURE 6:**
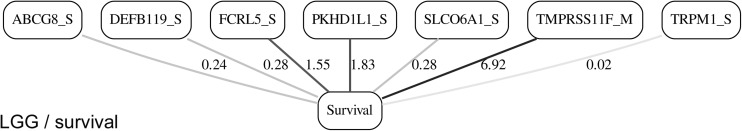
MN of the “Survival” node in the LGG/survival BN.

**FIGURE 7:**
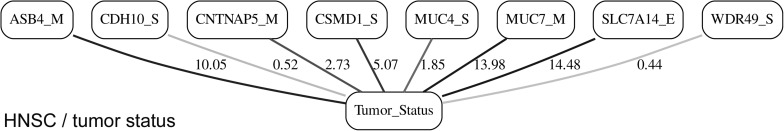
MN of the “Tumor_Status” node in the HNSC/tumor status BN.

**FIGURE 8:**
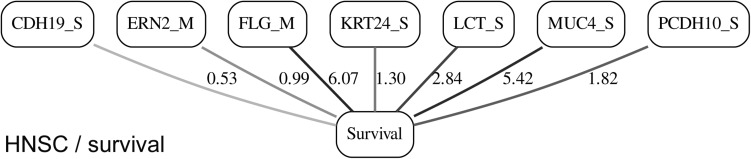
MN of the “Survival” node in the HNSC/survival BN.

**FIGURE 9:**
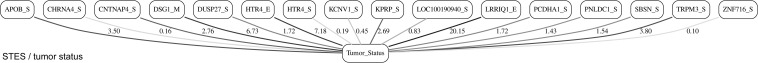
MN of the “Tumor_Status” node in the STES/tumor status BN.

**FIGURE 10:**
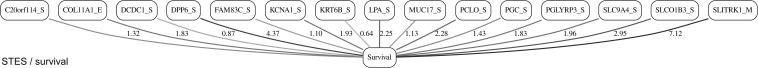
MN of the “Survival” node in the STES/survival BN.

While the resulting full BNs, in PDF format, are zoom-able and searchable, and the DOT files can be exported into the specialized network-oriented software, the full BNs tend to be visually overwhelming for the number of variables/nodes >100. Consequently, Figures 5–10 depict the immediate MNs of the clinical variables/nodes in the corresponding six BNs: LGG/tumor status (Figure 5), LGG/survival (Figure 6), HNSC/tumor status (Figure 7), HNSC/survival (Figure 8), STES/tumor status (Figure 9) and STES/survival (Figure 10).

It is noticeable in Figures 5–10 that all three molecular data components (S,E,and M) are represented in the MNs. This testifies to the efficacy and proportionality of both the MMI measure (during the variable selection stage) and the maximum entropy - based discretization (during the BN construction stage). Also of note, for some genes, more than one component is present (HTR4 E and S for STES/tumor status, CHIA E and S for LGG/tumor status, AFP E and S for LGG/tumor status). Conversely, some genes are associated with both tumor status and survival (MUC4 for HNSC, TMPRSS11F, SLC6A18, and DEFB119 for LGG).

**FIGURE 11:**
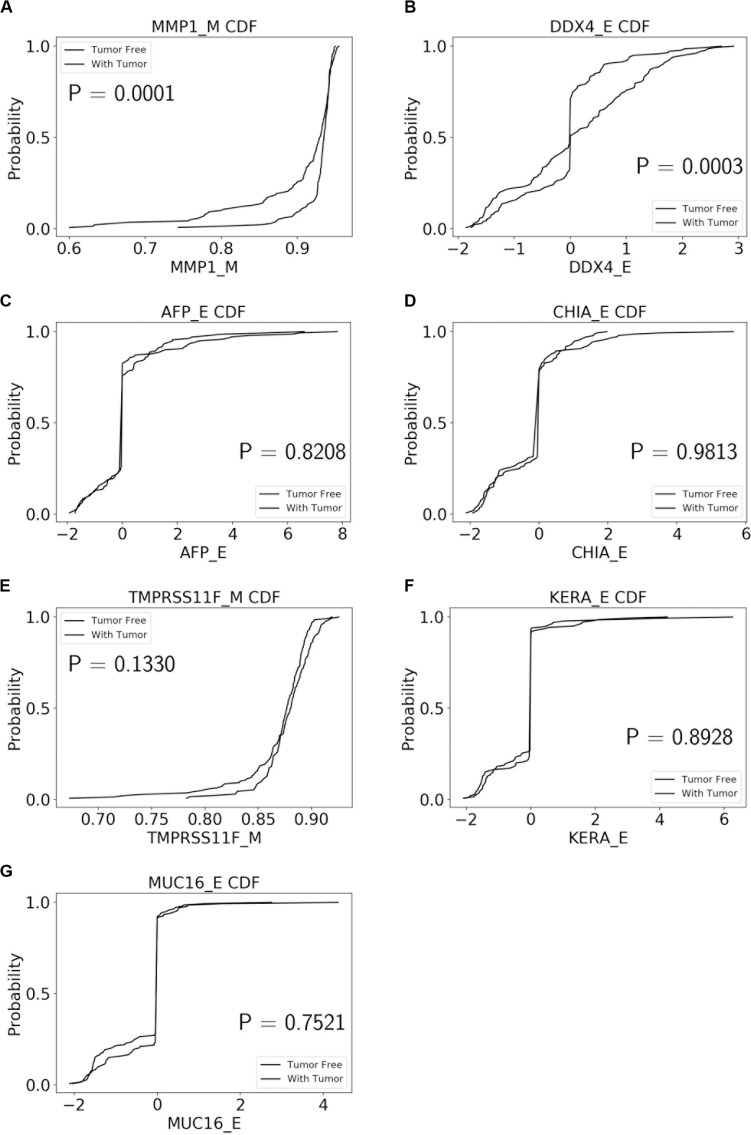
CDFs, shown separately for two “tumor status” groups, for seven continuous variables present in the MN depicted in Figure 5 (LGG/tumor status), in order of decreasing edge strength. **(A)** MMP1_M; **(B)** DDX4_E; **(C)** AFP_E; **(D)** CHIA_E; **(E)** TMPRSS11F_M; **(F)** KERA_E; **(G)** MUC16_E. *P-*values for the two-sample Kolmogorov-Smirnov test are shown in each chart.

The performance of our BN reconstruction algorithm or software is discussed in general terms in Gogoshin et al. (2017); here, we will evaluate the statistical significance of the resulting MNs. While the edge strength estimates in Figures 5–10 are useful in the relative sense, they do not immediately translate into the statistical significance measurements (such as *p-*values). Therefore, we have augmented the edge strengths with the *p-*values obtained *via* two-sample Kolmogorov–Smirnov (KS) probability distribution equality test (for continuous E and M molecular component variables) and two-sided Fisher’s exact test (for discrete S molecular component variable). To illustrate the KS test application, Figure 11 shows CDFs, separately for two “tumor status” groups, for seven continuous variables present in the MN depicted in Figure 5 (LGG/tumor status), in order of decreasing edge strength (Figure 11A, MMP1_M; Figure 11B, DDX4_E; Figure 11C, AFP_E; Figure 11D, CHIA_E; Figure 11E, TMPRSS11F_M; Figure 11F, KERA_E; Figure 11G, MUC16_E). Only MMP1_M and DDX4_E appear to be statistically highly significant, with TMPRSS11F_M being arguably a borderline case.

Table 1 lists the *p-*values for all 55 potentially predictive molecular gene components present in six MNs depicted in Figures 5–10, in order of decreasing edge strength for each network/MN. 12 gene components were found to be statistically significant (marked with an asterisk in Table 1), however, we decide to exclude LCT_S (marked with ^∗∗^ in Table 1) from further scrutiny because of the very low mutation counts in both survival groups.

**TABLE 1:** *P-*values for 55 potentially predictive molecular gene components present in six MNs depicted in Figures 5–10, subdivided by six datasets, in order of decreasing edge strength for each dataset/MN.

(A)		

Gene_component	BN edge strength	*p-*value
**LGG/tumor status**		
MMP1_M	14.13	0.0001*
DDX4_E	12.19	0.0003*
AFP_E	4.41	0.8208
CHIA_E	4.09	0.9813
TMPRSS11F_M	2.76	0.1330
KERA_E	2.23	0.8928
MUC16_E	0.66	0.7521
AFP_S	0.61	0.4985
SLC6A18_S	0.51	∼1.0
VIL1_S	0.45	0.2364
DEFB119_S	0.44	0.4872
C16orf11_S	0.41	∼1.0
CHIA_S	0.41	∼1.0

**(B)**		

**LGG/survival**		
TMPRSS11F_M	6.92	0.0003*
PKHD1L1_S	1.83	0.5818
FCRL5_S	1.55	0.1895
DEFB119_S	0.28	∼1.0
SLCO6A1_S	0.28	∼1.0
ABCG8_S	0.24	0.4366
TRPM1_S	0.02	0.1895

**(C)**		

**HNSC/tumor status**		
SLC7A14_E	14.48	2.1230e-05*
MUC7_M	13.98	0.3432
ASB4_M	10.05	0.2832
CSMD1_S	5.07	∼1.0
CNTNAP5_M	2.73	0.5261
MUC4_S	1.85	0.1496
CDH10_S	0.52	∼1.0
WDR49_S	0.44	∼1.0

**(D)**		

**HNSC/survival**		
FLG_M	6.07	0.2427
MUC4_S	5.42	0.4103
LCT_S	2.84	0.0226**
PCDH10_S	1.82	0.3544
KRT24_S	1.3	0.4942
ERN2_M	0.99	0.4832
CDH19_S	0.53	0.5787

**(E)**		

**STES/tumor status**		
LRRIQ_E	20.15	0.0004*
HTR4_E	7.18	0.2960
DSG1_M	6.73	0.1204
TRPM3_S	3.80	0.0245*
APOB_S	3.50	0.2723
CNTNAP4_S	2.76	0.7784
KPRP_S	2.69	∼1.0
DUSP27_S	1.72	0.5480
PCDHA1_S	1.72	0.8074
SBSN_S	1.54	0.6248
PNLDC1_S	1.43	∼1.0
LOC100190940_S	0.83	∼1.0
KCNV1_S	0.45	∼1.0
HTR4_S	0.19	0.3594
CHRNA4_S	0.16	0.3968
ZNF716_S	0.10	∼1.0

**(F)**		

**STES/survival**		
SLITRK1_M	7.12	0.1082
DPP6_S	4.37	0.0280*
SLCO1B3_S	2.95	0.0059*
PCLO_S	2.28	0.7238
LPA_S	2.25	0.0549
SLC9A4_S	1.96	0.0059*
KCNA1_S	1.93	0.0108*
COL11A1_E	1.83	0.2016
PGLYRP3_S	1.83	0.1119
PGC_S	1.43	0.4527
C20orf114_S	1.32	∼1.0
MUC17_S	1.13	0.0008*
FAM83C_S	1.10	0.6515
DCDC1_S	0.87	0.2051
KRT6B_S	0.64	∼1.0

Twelve gene components were found to be statistically significant (marked with *); LCT_S (marked with **) was excluded from further analysis because of the very low mutation counts (zero mutations in >2-year survival group, three mutations in <2-year survival group).

Subsequently, we performed manual literature/database search to ascertain if any of the remaining 11 genes were previously reported in the cancer context. The following resources were used: GeneCards (Stelzer et al., 2016) and DisGeNET (Pinero et al., 2017) databases, PubMed, and Google Scholar. Six genes were found to be implicated in cancer etiology/progression/clinical outcomes with high degree of certainty: MMP1, DDX4, TRPM3, DPP6, KCNA1, and MUC17 (Senapati et al., 2010; Saied et al., 2012; Lallet-Daher et al., 2013; Kawal et al., 2016; Park et al., 2016; Schudrowitz et al., 2017). Four genes (SLC7A14, LRRIQ, SLCO1B3, and SLC9A4) were supported by weaker, circumstantial evidence (Chan-On et al., 2013; Matullo et al., 2013; Fridley et al., 2016; Tanaka et al., 2017). One gene, TMPRSS11F, has not been discussed in the cancer context before, to the best of our knowledge [see also (Kataoka et al., 2018)]. However, increased expression levels of a similar type II transmembrane serine protease, TMPRSS11D, were found to be a significant non-small cell lung cancer survival predictor (Cao et al., 2017). Therefore, we suggest that TMPRSS11F should be further investigated as a strong predictive factor playing a role in LGG patients’ clinical characteristics – survival, especially. Lower TMPRSS11F methylation values correspond to a poorer long-term (2-year) survival. One possible mechanism is *via* the proteolysis of extracellular matrix which, in turn, is linked to the metastatic processes (Cao et al., 2017).

In summary, our analysis framework confirmed six well-known cancer-related genes, supplied additional evidence to support four other suspected cancer-related genes, and identified one novel potentially strongly predictive factor, methylation of TMPRSS11F.

## Discussion

Systems biology approach to the complex genetic and epigenetic cancer data analysis is arguably superior to the simpler single-gene (or even single-data type) alternatives. However, it is intrinsically linked to the fundamental, interrelated, challenges – scalability, “curse of dimensionality,” accounting for non-additive, higher-order interactions, and visualization of the results (i.e., translation of the massive network graphs into concrete biomedical insights). In this study we propose a flexible and generalizable approach to the BN-based systems biology analysis of the multi-modal cancer data, using the TCGA database as an example. It consists of the variable selection step (which is not computationally demanding) and the BN reconstruction step (which is substantially computationally demanding). Ideally, the investigators would simply feed the complete dataset (all variables) into the BN software, obtain the full graphical model (no matter how large and complex), and then “zoom in” on the MN of the variable(s) of interest, such as a clinical outcome or a cancer phenotype. However, this is impractical for most real datasets and available hardware configurations.

Consequently, we propose starting with the variable selection step to select a (relatively) small subset of genes that are associated with the variable(s) of interest (tumor status and 2-year survival in the present study). The principal novelty of our approach lies in using the MMI measure for the variable/gene selection, in which all possible types of molecular information (discrete and continuous, genetic and epigenetic) are considered simultaneously. The other innovative aspect of our approach lies in the adjustability of the “hand-off” point between the variable selection and BN modeling steps. This hand-off point can depend on the investigators’ computational resources, the shape of the variable selection curves, or the predefined statistical cutoff points. For example, ∼20 K genes can be reduced to 100–200 genes for the subsequent BNs construction, in which case the complete analysis takes less than an hour on a mid-level PC. When feeding the complete datasets (10,000–15,000 genes, in case of TCGA and similar genomic resources) into our BN software (Gogoshin et al., 2017), without the preliminary variable selection step, it takes about 3 days to build a full BN on a dedicated multi-core workstation. Therefore, the investigators can choose the appropriate balance depending on whether they are interested in a quick, exploratory analysis or a finalized, exhaustive one.

In our analyses, the final predictive gene sets (such as shown in Table 1) were different from the sets (of comparable sizes) of “top” genes obtained in the variable selection step alone (otherwise there would be no need to invoke the computationally expensive BN modeling step). This was to be expected, because BN modeling is a multivariate modeling tool (which aims to reconstruct the most fitting pattern of conditional independencies in the MN of a clinical variable), while MMI ranking is a univariate variable selection “filter” that does not account for the dependencies between the (top) genes. Another reason that the two corresponding gene sets tend to be different has to do with the fact that the first analysis stage is gene-centric, whereas the second analysis stage separates the three molecular modalities. Limiting our analysis pipeline to just the first stage (MMI filter/ranking) would therefore miss the strong one-modality (but week multiple-modalities) predictors. In future, we plan to study the extent of intersection of such two sets as a function of the “hand-off” point (between MMI pre-ranking and full BN analysis) parameter.

Our computational pipeline is inherently generalizable, as it can be directly applied to any large multimodal genetic/epigenetic dataset with minimal preprocessing. The only two changeable parameters are the aforementioned variable selection/BN modeling hand-off point, and the BN discretization mechanism. The latter is currently set as the 3-bin maximum entropy-based discretization coupled with the multinomial local probability model (Gogoshin et al., 2017). This is not the most elegant, or universally applicable, solution. In future, we plan to develop a novel BN model scoring function derived from a mixed distance measure (such as the MMI), or a similar metric that expresses divergence between the current network model and the data *via* mixed-type distances. The resulting two-stage analytical strategy will thus fully automatically deal with the mixed variables, in both of its stages. This has not been done before, so we plan to implement and test the MMI-based BN algorithm alongside the more established mixed-type BN solutions (hybrid local probability models, adaptive discretization), and use both real and simulated data to investigate which method is preferable.

Another limitation of the present study has to do with its primary focus on the clinical outcomes/phenotypes; at this time, we decided to largely concentrate on the MNs of the clinical variables/nodes. In future, we intend to analyze the resulting full BNs more “holistically,” paying attention to the general network topological properties, gene clusters, hub and bottleneck genes, etc. Consequently, one useful extension of our analytical framework would be to incorporate multiple clinical outcomes/phenotypes into the network analyses, to see if the inter-outcome dependencies are reflected in the resulting networks, and if they are mediated by other nodes/variables.

Application of our pipeline to TCGA data resulted in the identification of a number of candidate genes for the different clinical cancer characteristics, *via* varied molecular components. It is well known that epigenetic processes/DNA methylation play an important role in many cancers’ diagnosis, progression, and outcome; our results support that notion, as many of the most statistically significant predictors generated in the present study were in fact the methylation molecular components (Table 1). Notably, the one novel candidate gene pinpointed in this study, TMPRSS11F, likely would not have been identified *via* any other (non-epigenetic) modality. Our results, therefore, underscore the essentiality of the simultaneous analysis of different molecular modalities, including the epigenetic ones, for the precision or personalized medicine to be effective in cancer treatment.

## Data Availability Statement

All the intermediate datasets generated for this study are available on request to the corresponding author.

## Author Contributions

XW, SB, and AR conceptualized the study, carried out the analyses, and wrote the manuscript. GG and SD contributed to carrying out the analyses. All authors contributed to the article and approved the submitted version.

## Conflict of Interest

The authors declare that the research was conducted in the absence of any commercial or financial relationships that could be construed as a potential conflict of interest.

## Supplementary Material

TABLES S1–S6 Data files, in Excel format, listing the genes analyzed in this study, together with the corresponding MMI values, in order of decreasing MMI values. **(1)** LGG/tumor status, **(2)** LGG/survival, **(3)** HNSC/tumor status, **(4)** HNSC/survival, **(5)** STES/tumor status, **(6)** STES/survival. Genes with the MMI values >99.5% MMI CDF (i.e., genes selected for further BN analyses) are shown in bold.

TABLES S7–S12 Full Bayesian networks, in Word/DOT format, for the six datasets in this study. **(1)** LGG/tumor status, **(2)** LGG/survival, **(3)** HNSC/tumor status, **(4)** HNSC/survival, **(5)** STES/tumor status, **(6)** STES/survival.

DATA SHEETS S1–S6 Full Bayesian networks, in PDF format, for the six datasets in this study. **(1)** LGG/tumor status, **(2)** LGG/survival, **(3)** HNSC/tumor status, **(4)** HNSC/survival, **(5)** STES/tumor status, **(6)** STES/survival. Designations are as in main text Figure 3.

## References

[B1] Andrews B. Ramsey J. Cooper G. F. (2018). Scoring Bayesian Networks of Mixed Variables. *Int. J. Data Sci. Anal.* 6 3–18. 10.1007/s41060-017-0085-7 30140730 PMC6101981

[B2] Asur S. Ucar D. Parthasarathy S. (2007). An ensemble framework for clustering protein-protein interaction networks. *Bioinformatics* 23 i29–i40. 10.1093/bioinformatics/btm212 17646309

[B3] Blum A. L. Langley P. (1997). Selection of relevant features and examples in machine learning. *Artif. Intell.* 97 245–271. 10.1016/s0004-3702(97)00063-5

[B4] Cao X. Tang Z. Huang F. Jin Q. Zhou X. Shi J. (2017). High TMPRSS11D protein expression predicts poor overall survival in non-small cell lung cancer. *Oncotarget* 8 12812–12819. 10.18632/oncotarget.14559 28086212 PMC5355057

[B5] Chan-On W. Nairismagi M. L. Ong C. K. Lim W. K. Dima S. Pairojkul C. (2013). Exome sequencing identifies distinct mutational patterns in liver fluke-related and non-infection-related bile duct cancers. *Nat. Genet.* 45 1474–1478. 10.1038/ng.2806 24185513

[B6] Fang X. Yin Z. Li X. Xia L. Quan X. Zhao Y. (2017). Multiple functional SNPs in differentially expressed genes modify risk and survival of non-small cell lung cancer in chinese female non-smokers. *Oncotarget* 8 18924–18934. 10.18632/oncotarget.14836 28148898 PMC5386658

[B7] Fridley B. L. Ghosh T. M. Wang A. Raghavan R. Dai J. Goode E. L. (2016). Genome-wide study of response to platinum. taxane, and combination therapy in ovarian cancer: in vitro phenotypes, inherited variation, and disease recurrence. *Front Genet* 7:37. 10.3389/fgene.2016.00037 27047539 PMC4801852

[B8] Friedman N. Linial M. Nachman I. Pe’er D. (2000). Using Bayesian networks to analyze expression data. *J. Comput. Biol.* 7 601–620. 10.1089/106652700750050961 11108481

[B9] Gao W. Kannan S. Oh S. Viswanath P. (2018). Estimating mutual information for discrete-continuous mixtures. *arXiv [Preprint].* Available online at: https://arxiv.org/abs/1709.06212

[B10] Gevaert O. De Smet F. Timmerman D. Moreau Y. De Moor B. (2006). Predicting the prognosis of breast cancer by integrating clinical and microarray data with Bayesian networks. *Bioinformatics* 22 e184–e190. 10.1093/bioinformatics/btl230 16873470

[B11] Gogoshin G. Boerwinkle E. Rodin A. S. (2017). New Algorithm and Software (BNOmics) for Inferring and Visualizing Bayesian Networks from Heterogeneous Big Biological and Genetic Data. *J. Comput. Biol.* 24 340–356. 10.1089/cmb.2016.0100 27681505 PMC5372779

[B12] Guyon I. Elisseeff A. (2003). An introduction to variable and feature selection. *J. Mach. Learn. Res.* 3 1157–1182.

[B13] Heckerman D. (1995). *Tutorial on Learning with Bayesian Networks.* Technical Report MSR-TR-95–96 Redmond, WA: Microsoft Research.

[B14] Hou X. He X. Wang K. Hou N. Fu J. (2018). Genome-wide network-based analysis of colorectal cancer identifies novel prognostic factors and an integrative prognostic index. *Cell Physiol. Biochem.* 49 1703–1716. 10.1159/000493614 30248669

[B15] Huang T. Yang J. Cai Y. D. (2015). Novel candidate key drivers in the integrative network of genes, microRNAs, methylations, and copy number variations in squamous cell lung carcinoma. *Biomed. Res. Int.* 2015 358125. 10.1155/2015/358125 PMC4352729 25802847

[B16] Jeong H. Leem S. Wee K. Sohn K. A. (2015). Integrative network analysis for survival-associated gene-gene interactions across multiple genomic profiles in ovarian cancer. *J. Ovarian. Res.* 8:42. 10.1186/s13048-015-0171-1 PMC4491426 26138921

[B17] Kaiser J. L. Bland C. L. Klinke D. J. II (2016). Identifying causal networks linking cancer processes and anti-tumor immunity using Bayesian network inference and metagene constructs. *Biotechnol. Prog.* 32 470–479. 10.1002/btpr.2230 26785356 PMC5289651

[B18] Kataoka H. Kawaguchi M. Fukushima T. Shimomura T. (2018). Hepatocyte growth factor activator inhibitors (HAI-1 and HAI-2): emerging key players in epithelial integrity and cancer. *Pathol. Int.* 68 145–158. 10.1111/pin.12647 29431273

[B19] Kawal P. Chandra A. Rajkumar Dhole T. N. Ojha B. (2016). Correlations of polymorphisms in matrix metalloproteinase-1, -2, and -7 promoters to susceptibility to malignant gliomas. *Asian J. Neurosurg.* 11 160–166. 27057223 10.4103/1793-5482.145338 PMC4802938

[B20] Koch A. Joosten S. C. Feng Z. de Ruijter T. C. Draht M. X. Melotte V. (2018). Analysis of DNA methylation in cancer: location revisited. *Nat. Rev. Clin. Oncol.* 15 459–466. 10.1038/s41571-018-0004-4 29666440

[B21] Kohavi R. John G. H. (1997). Wrappers for feature subset selection. *Artif. Intell.* 97 273–324. 10.1016/s0004-3702(97)00043-x

[B22] Kraskov A. Stögbauer H. Grassberger P. (2004). Estimating mutual information. *Phys. Rev. E* 69 066138. 10.1103/PhysRevE.69.066138 15244698

[B23] Lallet-Daher H. Wiel C. Gitenay D. Navaratnam N. Augert A. Calvé B. L. (2013). Potassium channel KCNA1 modulates oncogene-induced senescence and transformation. *Cancer Res.* 73 5253–5265. 10.1158/0008-5472.can-12-3690 23774215

[B24] Leng J. Valli C. Armstrong L. (2010). “A wrapper-based feature selection for analysis of large data sets,” in *Proceedings of the Conference: 3rd International Conference on Computer and Electrical Engineering (ICCEE 2010)*, Chengdu.

[B25] Liang A. Zhou B. Sun W. (2017). Integrated genomic characterization of cancer genes in glioma. *Cancer Cell Int.* 17:90. 10.1186/s12935-017-0458-y PMC5640938 29046615

[B26] Margolin A. A. Nemenman I. Basso K. Wiggins C. Stolovitzky G. Favera R. D. (2006). ARACNE: an algorithm for the reconstruction of gene regulatory networks in a mammalian cellular context. *BMC Bioinformatics* 7 (Suppl. 1):S7. 10.1186/1471-2105-7-S1-S7 16723010 PMC1810318

[B27] Matullo G. Guarrera S. Betti M. Fiorito G. Ferrante D. Voglino F. (2013). Genetic variants associated with increased risk of malignant pleural mesothelioma: a genome-wide association study. *PLoS One* 8:e61253. 10.1371/journal.pone.0061253 23626673 PMC3634031

[B28] Park Y. R. Chun J. N. So I. Kim H. J. Baek S. Jeon J. H. (2016). Data-driven Analysis of TRP channels in cancer: linking variation in gene expression to clinical significance. *Cancer Genom. Proteom.* 13 83–90. 26708603

[B29] Phan J. H. Hoffman R. Kothari S. Wu P. Y. Wang M. D. (2016). Integration of multi-modal biomedical data to predict cancer grade and patient survival. *IEEE EMBS Int. Conf. Biomed. Health Inform.* 2016 577–580. 27493999 10.1109/BHI.2016.7455963 PMC4969000

[B30] Phipps A. I. Passarelli M. N. Chan A. T. Harrison T. A. Jeon J. Hutter C. M. (2016). Common genetic variation and survival after colorectal cancer diagnosis: a genome-wide analysis. *Carcinogenesis* 37 87–95. 26586795 10.1093/carcin/bgv161 PMC4715234

[B31] Pinero J. Bravo A. Queralt-Rosinach N. Gutierrez-Sacristan A. Deu-Pons J. Centeno E. (2017). DisGeNET: a comprehensive platform integrating information on human disease-associated genes and variants. *Nucleic Acids Res.* 45 D833–D839. 27924018 10.1093/nar/gkw943 PMC5210640

[B32] Rajesh Y. Pal I. Banik P. Chakraborty S. Borkar S. A. Dey G. (2017). Insights into molecular therapy of glioma: current challenges and next generation blueprint. *Acta Pharmacol. Sin.* 38 591–613. 10.1038/aps.2016.167 28317871 PMC5457688

[B33] Ramsey J. Glymour M. Sanchez-Romero R. Glymour C. (2017). A million variables and more: the Fast Greedy Equivalence Search algorithm for learning high-dimensional graphical causal models, with an application to functional magnetic resonance images. *Int. J. Data Sci. Anal.* 3 121–129. 10.1007/s41060-016-0032-z 28393106 PMC5380925

[B34] Rodin A. S. Litvinenko A. Klos K. Morrison A. C. Woodage T. Coresh J. (2009). Use of wrapper algorithms coupled with a random forests classifier for variable selection in large-scale genomic association studies. *J. Comput. Biol.* 16 1705–1718. 10.1089/cmb.2008.0037 20047492 PMC2980837

[B35] Saied M. H. Marzec J. Khalid S. Smith P. Down T. A. Rakyan V. K. (2012). Genome wide analysis of acute myeloid leukemia reveal leukemia specific methylome and subtype specific hypomethylation of repeats. *PLoS One* 7:e33213. 10.1371/journal.pone.0033213 22479372 PMC3315563

[B36] Schudrowitz N. Takagi S. Wessel G. M. Yajima M. (2017). Germline factor DDX4 functions in blood-derived cancer cell phenotypes. *Cancer Sci.* 108 1612–1619. 10.1111/cas.13299 28612512 PMC5543511

[B37] Sedgewick A. J. Buschur K. Shi I. Ramsey J. D. Raghu V. K. Manatakis D. V. (2018). Mixed graphical models for integrative causal analysis with application to chronic lung disease diagnosis and prognosis. *Bioinformatics* 35 1204–1212. 10.1093/bioinformatics/bty769 30192904 PMC6449754

[B38] Senapati S. Ho S. B. Sharma P. Das S. Chakraborty S. Kaur S. (2010). Expression of intestinal MUC17 membrane-bound mucin in inflammatory and neoplastic diseases of the colon. *J. Clin. Pathol.* 63 702–707. 10.1136/jcp.2010.078717 20702471 PMC2997570

[B39] Stelzer G. Rosen N. Plaschkes I. Zimmerman S. Twik M. Fishilevich S. (2016). The genecards suite: from gene data mining to disease genome sequence analyses. *Curr. Protoc. Bioinformatics* 54:1.30.1-1.30.33. 10.1002/cpbi.5 27322403

[B40] Tanaka H. Kanda M. Shimizu D. Tanaka C. Kobayashi D. Hayashi M. (2017). FAM46C serves as a predictor of hepatic recurrence in patients with resectable gastric cancer. *Ann. Surg. Oncol.* 24 3438–3445. 10.1245/s10434-016-5636-y 27770343

[B41] Tian W. Li Y. Zhang J. Li J. Gao J. (2018). Combined analysis of DNA methylation and gene expression profiles of osteosarcoma identified several prognosis signatures. *Gene* 650 7–14. 10.1016/j.gene.2018.01.093 29407229

[B42] Tomczak K. Czerwinska P. Wiznerowicz M. (2015). The Cancer Genome Atlas (TCGA): an immeasurable source of knowledge. *Contemp. Oncol.* 19 A68–A77. 10.5114/wo.2014.47136 PMC4322527 25691825

[B43] Wang W. Baladandayuthapani V. Holmes C. C. Do K. A. (2013). Integrative network-based Bayesian analysis of diverse genomics data. *BMC Bioinformatics* 14 (Suppl. 13):S8. 10.1186/1471-2105-14-S13-S8 24267288 PMC3849715

[B44] Wu S. Law A. Whipple M. E. (2017). A bayesian network model of head and neck squamous cell carcinoma incorporating gene expression profiles. *Stud. Health Technol. Inform.* 245 634–638. 29295173

[B45] Xu P. Yang J. Liu J. Yang X. Liao J. Yuan F. (2018). Identification of glioblastoma gene prognosis modules based on weighted gene co-expression network analysis. *BMC Med. Genomics* 11:96. 10.1186/s12920-018-0407-1 30382873 PMC6211550

[B46] Xu Y. Zhang J. Yuan Y. Mitra R. Muller P. Ji Y. (2012). A bayesian graphical model for integrative analysis of TCGA Data. *IEEE Int. Workshop Genomic Signal Process. Stat.* 2012 135–138. 25859418 10.1109/GENSIPS.2012.6507747 PMC4387199

[B47] Xu Y. Zhu Y. Muller P. Mitra R. Ji Y. (2014). Characterizing Cancer-Specific Networks by Integrating TCGA Data. *Cancer Inform.* 13 125–131. 10.4137/CIN.S13776 PMC4657757 26628858

[B48] Yoo B. C. Kim K. H. Woo S. M. Myung J. K. (2017). Clinical multi-omics strategies for the effective cancer management. *J. Proteomics* 188 97–106. 10.1016/j.jprot.2017.08.010 28821459

[B49] Zhang Q. Burdette J. E. Wang J. P. (2014). Integrative network analysis of TCGA data for ovarian cancer. *BMC Syst Biol* 8:1338. 10.1186/s12918-014-0136-9 25551281 PMC4331442

[B50] Zhang X. Branciamore S. Gogoshin G. Rodin A. S. Riggs A. D. (2017). Analysis of high-resolution 3D intrachromosomal interactions aided by Bayesian network modeling. *Proc. Natl. Acad. Sci. U.S.A.* 114 E10359–E10368. 29133398 10.1073/pnas.1620425114 PMC5715735

[B51] Zhang C. Cheng W. Ren X. Wang Z. Liu X. Li G. (2017). Tumor purity as an underlying key factor in Glioma. *Clin. Cancer Res.* 23 6279–6291. 10.1158/1078-0432.ccr-16-2598 28754819

[B52] Zhu Y. Xu Y. Helseth D. L. Jr. Gulukota K. Yang S. Pesce L. L. (2015). Zodiac: a comprehensive depiction of genetic interactions in cancer by integrating TCGA data. *J. Natl. Cancer Inst.* 107:djv129. 10.1093/jnci/djv129 PMC4554190 25956356

