# Supplementary figures and images for: New Analysis Framework Incorporating Mixed Mutual Information and Scalable Bayesian Networks for Multimodal High Dimensional Genomic and Epigenomic Cancer Data
Source: Front Genet. 2020 Jun 18;11:648. doi: 10.3389/fgene.2020.00648 (PMC7314938; doi:10.3389/fgene.2020.00648)

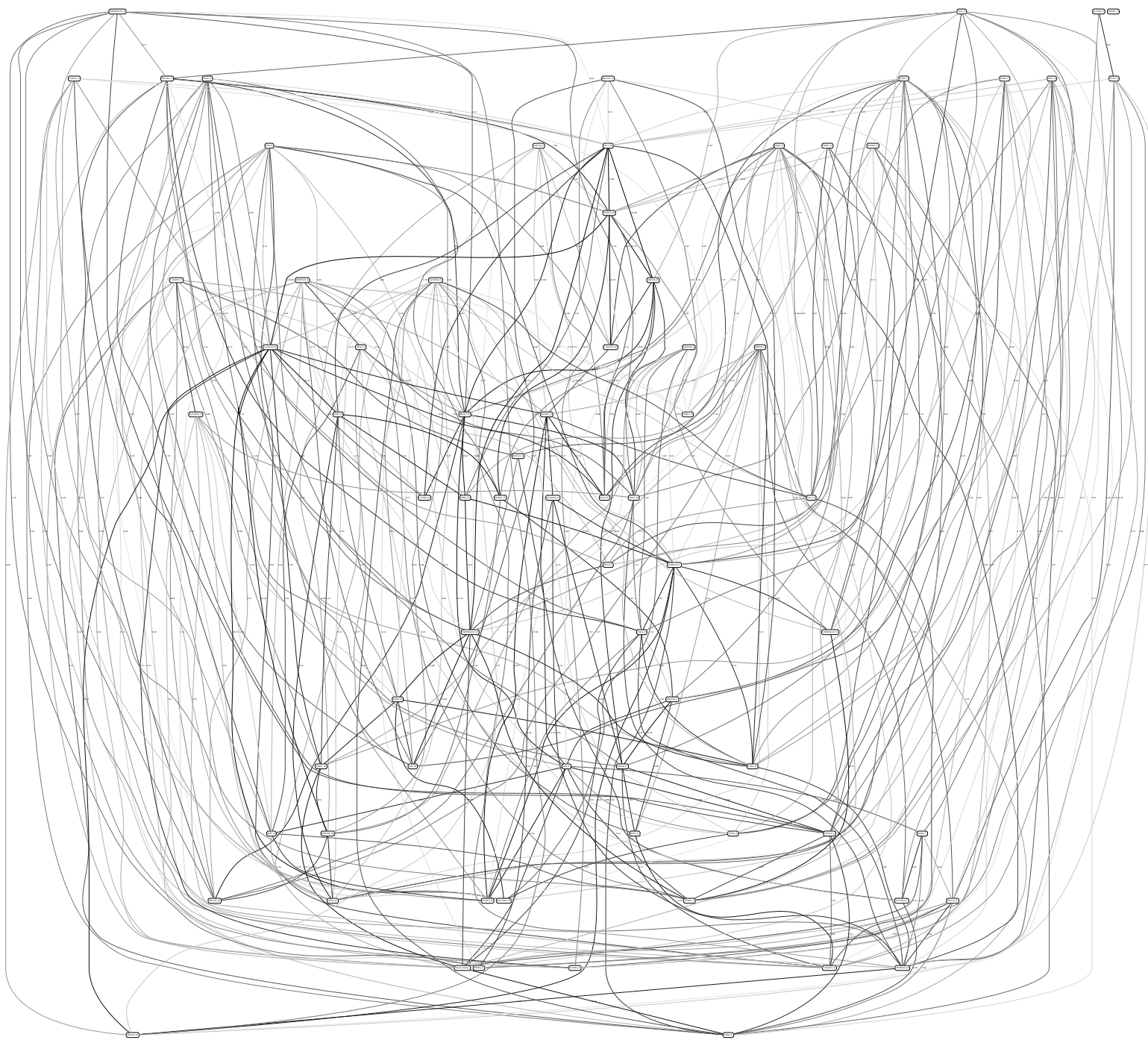

Supplement: DATA SHEETS S1–S6 — Full Bayesian networks, in PDF format, for the six datasets in this study. (1) LGG/tumor status, (2) LGG/survival, (3) HNSC/tumor status, (4) HNSC/survival, (5) STES/tumor status, (6) STES/survival. Designations are as in main text Figure 3. [file Data_Sheet_1.PDF]

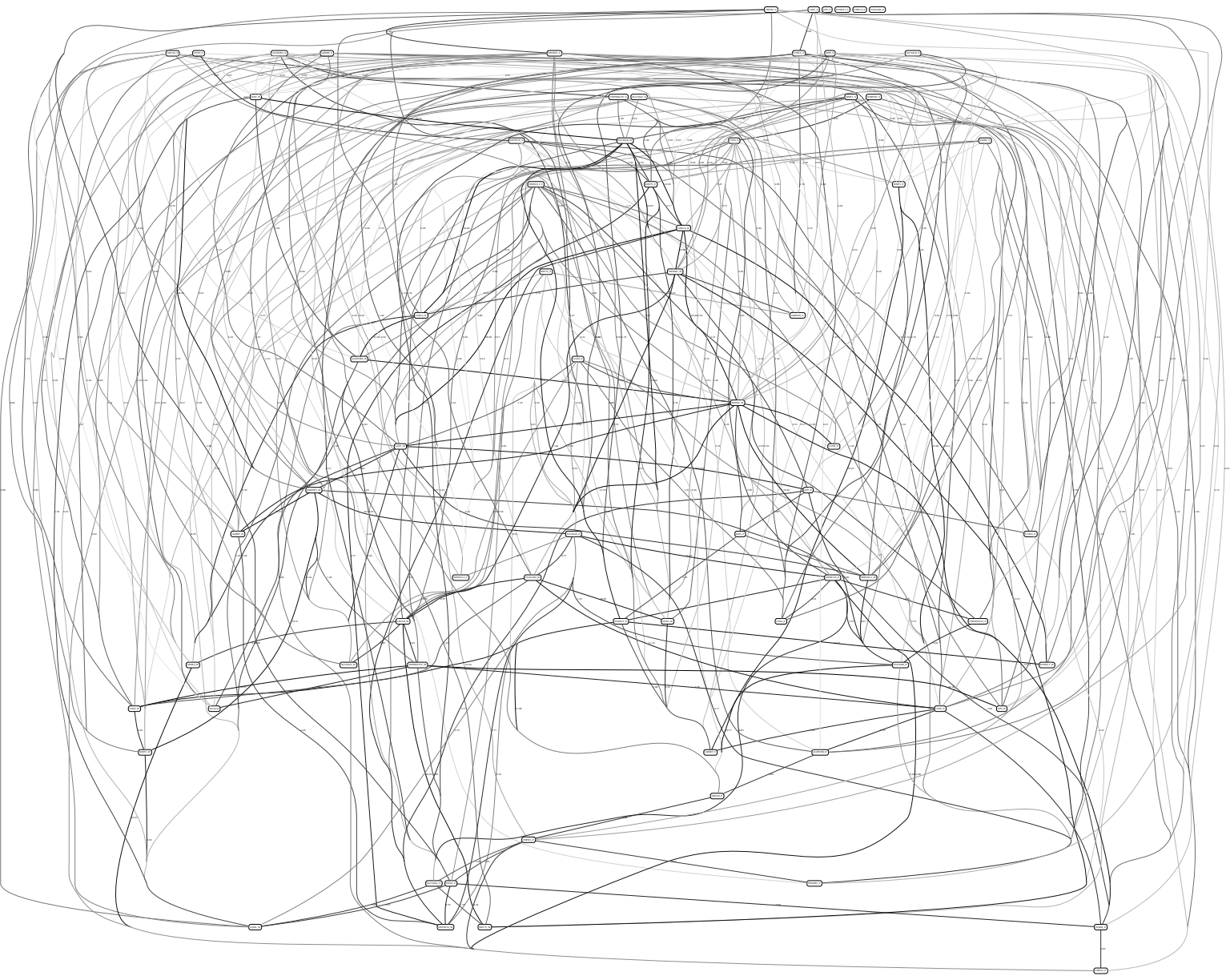

Supplement: Supplementary file 14 [file Data_Sheet_2.PDF]

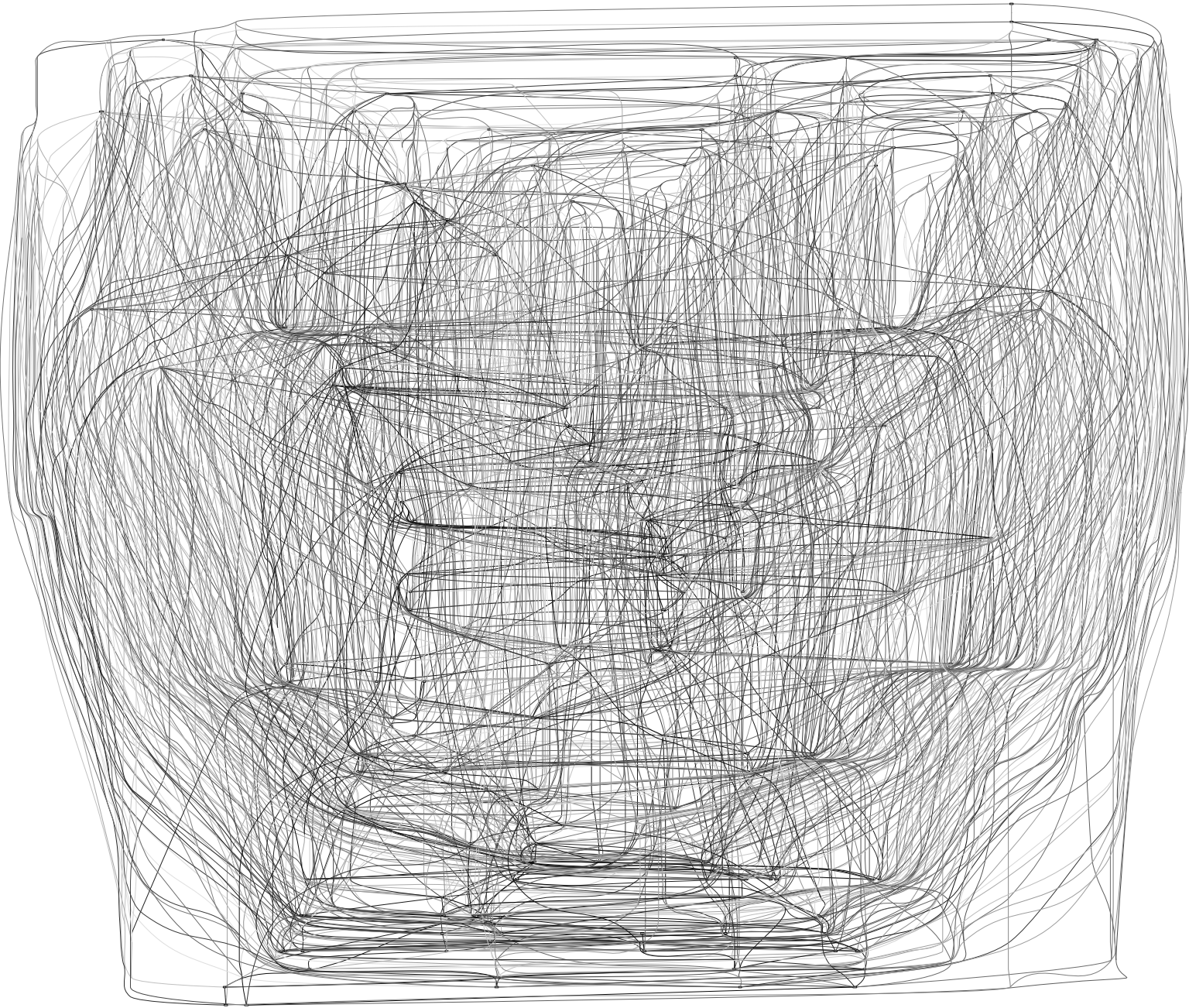

Supplement: Supplementary file 16 [file Data_Sheet_4.PDF]

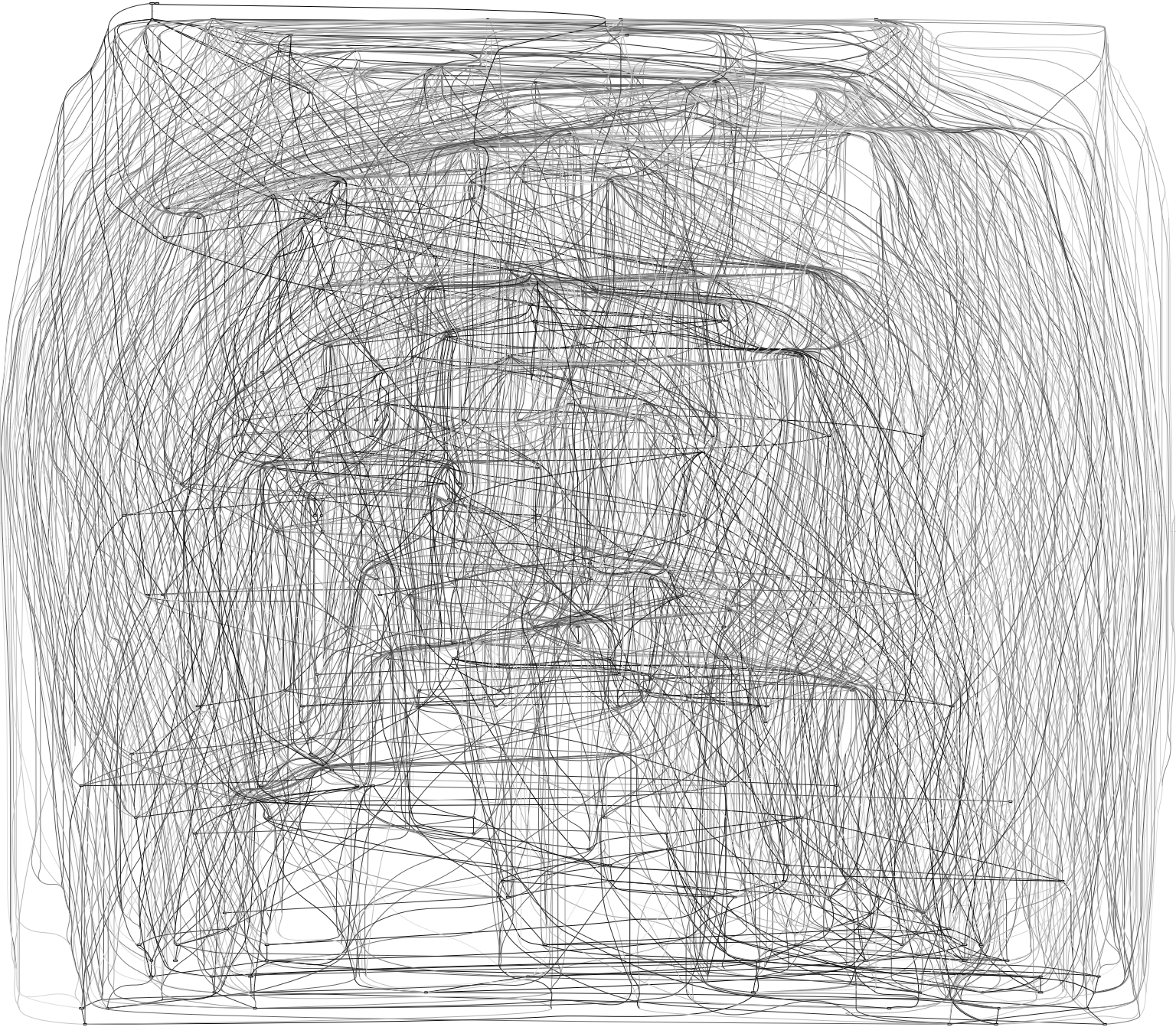

Supplement: Supplementary file 17 [file Data_Sheet_5.PDF]

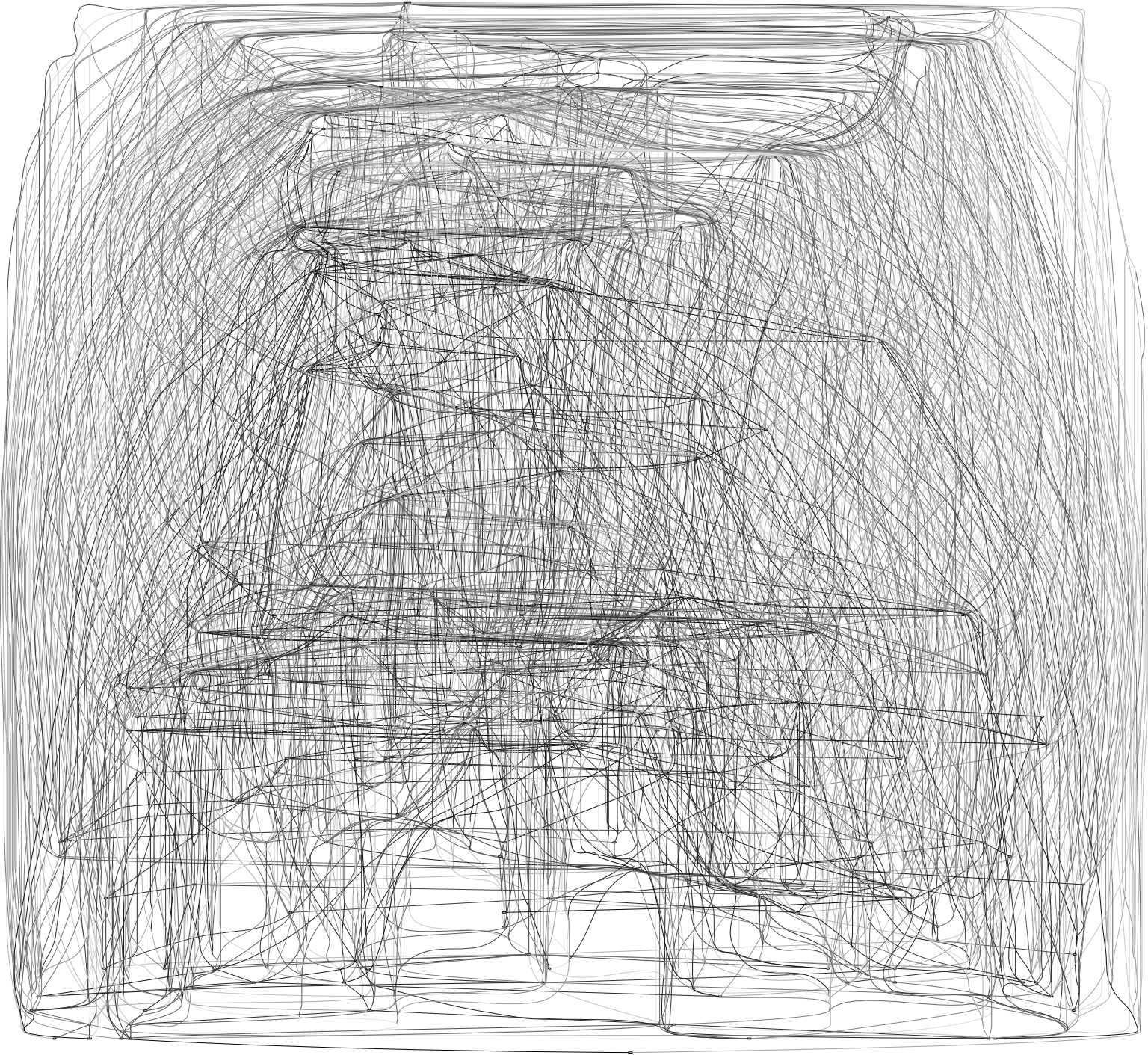

Supplement: Supplementary file 18 [file Data_Sheet_6.PDF]
